# Cardiovascular Benefit of Empagliflozin Across the Spectrum of Cardiovascular Risk Factor Control in the EMPA-REG OUTCOME Trial

**DOI:** 10.1210/clinem/dgaa321

**Published:** 2020-06-02

**Authors:** Silvio E Inzucchi, Kamlesh Khunti, David H Fitchett, Christoph Wanner, Michaela Mattheus, Jyothis T George, Anne Pernille Ofstad, Bernard Zinman

**Affiliations:** 1 Section of Endocrinology, Yale School of Medicine, New Haven, Connecticut; 2 Diabetes Research Centre, University of Leicester, Leicester General Hospital, Leicester, UK; 3 Division of Cardiology, St. Michael’s Hospital, University of Toronto, Toronto, Ontario, Canada; 4 Division of Nephrology, Würzburg University Clinic, Würzburg, Germany; 5 Boehringer Ingelheim Pharma GmbH & Co.KG, Ingelheim, Germany; 6 Boehringer Ingelheim International GmbH, Ingelheim, Germany; 7 Boehringer Ingelheim Norway KS, Asker, Norway; 8 Lunenfeld-Tanenbaum Research Institute, Mount Sinai Hospital, University of Toronto, Toronto, Ontario, Canada

**Keywords:** type 2 diabetes, cardiovascular disease, cardioprotective

## Abstract

**Context:**

Control of multiple cardiovascular (CV) risk factors reduces CV events in individuals with type 2 diabetes.

**Objective:**

To investigate this association in a contemporary clinical trial population, including how CV risk factor control affects the CV benefits of empagliflozin, a sodium-glucose cotransporter-2 inhibitor.

**Design:**

Post hoc analysis.

**Setting:**

Randomized CV outcome trial (EMPA-REG OUTCOME).

**Participants:**

Type 2 diabetes patients with established CV disease.

**Intervention:**

Empagliflozin or placebo.

**Main Outcome Measures:**

Risk of CV outcomes—including the treatment effect of empagliflozin—by achieving 7 goals for CV risk factor control at baseline: (1) glycated hemoglobin <7.5%, (2) low-density lipoprotein cholesterol <100 mg/dL or statin use, (3) systolic blood pressure <140 mmHg and diastolic blood pressure <90 mmHg, (4) pharmacological renin-angiotensin-aldosterone system blockade, (5) normoalbuminuria, (6) aspirin use, (7) nonsmoking.

**Results:**

In the placebo group, the hazard ratio (HR) for CV death was 4.00 (95% CI, 2.26–7.11) and 2.48 (95% CI, 1.52–4.06) for patients achieving only 0–3 or 4–5 risk factor goals at baseline, respectively, compared with those achieving 6–7 goals. Participants achieving 0–3 or 4–5 goals also had increased risk for the composite outcome of hospitalization for heart failure or CV death (excluding fatal stroke) (HR 2.89 [1.82–4.57] and 1.90 [1.31–2.78], respectively) and 3-point major adverse CV events (HR 2.21 [1.53–3.19] and 1.42 [1.06–1.89]). Empagliflozin significantly reduced these outcomes across all risk factor control categories (*P* > 0.05 for treatment-by-subgroup interactions).

**Conclusions:**

Cardiovascular risk in EMPA-REG OUTCOME was inversely associated with baseline CV risk factor control. Empagliflozin’s cardioprotective effect was consistent regardless of multiple baseline risk factor control.

Type 2 diabetes (T2D) is an increasingly prevalent condition and a major cause of atherosclerotic cardiovascular (CV) disease (ASCVD) (1, 2). The excess risk for ASCVD in patients with T2D has been estimated to be at least twice that of those without diabetes (3–5), although it may be lower in contemporary cohorts receiving modern CVD preventive care (6). Numerous studies have demonstrated that isolated control of modifiable CV risk factors such as elevated blood pressure or low-density lipoprotein (LDL) cholesterol reduces ASCVD risk in people with T2D (1). However, despite clinical guidelines recommending multifactorial intervention (1), evidence supporting simultaneous control of multiple cardiometabolic risk factors in patients with diabetes is somewhat limited. The randomized Steno-2 trial found the long-term risk for CV events was halved in patients with T2D and microalbuminuria receiving multifactorial intensive treatment for hyperglycemia, hypertension, and dyslipidemia (7). More recent randomized trials of intensified multifactorial treatment in Japan and Europe, however, had neutral findings as compared with the standard of care (8, 9). To date, most studies supporting multifactorial intervention for ASCVD risk reduction in T2D are observational in nature (10–19).

Diabetes also increases the risk for heart failure (1, 2, 20), which some recent studies have found to be a more common complication in T2D than ASCVD (20–22). The awareness of heart failure as an important complication in T2D re-emerged in recent years following the finding that certain glucose-lowering drugs increased the risk of hospitalization for this condition (20). Recent studies have found that the age- and sex-adjusted rate of heart failure hospitalizations is approximately twice as high in individuals with diabetes as in those without (23, 24). Furthermore, simultaneous control of multiple risk factors appears to reduce—but not eliminate—the excess risk of hospitalization for heart failure in patients with T2D (17).

Although male sex is associated with worse CV outcomes (25), women are less likely to have CV risk factors measured, and younger women may receive less aggressive CV risk factor management than their male counterparts (26). Data from the Swedish Heart Failure Registry showed that among patients with T2D and heart failure, women also received less guideline-recommended treatment for heart failure and carried a worse prognosis than men (27).

Empagliflozin is a sodium-glucose cotransporter-2 (SGLT2) inhibitor used as an adjunct to diet and exercise to improve glycemic control in adults with T2D. In the landmark EMPA-REG OUTCOME trial, empagliflozin became the first glucose-lowering drug to demonstrate a reduced risk for CV events in high-risk patients with T2D (28), as a result of which it also became the first to earn a label indication for reducing CV mortality. In this CV outcome trial in patients with T2D and established ASCVD, empagliflozin added to the standard of care significantly reduced the risk of CV death by 38% (28). In addition, empagliflozin significantly reduced the risk of heart failure hospitalization by 35% and major adverse CV events (CV death, nonfatal myocardial infarction [MI], or nonfatal stroke [3-point major adverse cardiovascular events (3P-MACE)]) by 14% (28), with similar effects in men and women (29).

In order to explore the relationship between simultaneous control of multiple modifiable risk factors and CV outcomes, including hospitalization for heart failure, in people with T2D in a contemporary setting, we compared the risk for outcomes in the EMPA-REG OUTCOME trial across different categories of risk factor control at baseline. We also sought to determine whether the CV benefits of empagliflozin might be influenced by the underlying control of CV risk factors, and if this differed between men and women.

## Materials and Methods

The study design of the EMPA-REG OUTCOME trial is described in detail elsewhere (28, 30). In brief, after randomization, 7020 individuals aged ≥18 years with T2D, glycated hemoglobin (HbA1c) 7% to 10% (53–86 mmol/mol), established ASCVD, body-mass index (BMI) ≤45 kg/m^2^, and an estimated glomerular filtration rate (eGFR) ≥30 mL/min/1.73 m2 received double-blind treatment with empagliflozin 10 mg, empagliflozin 25 mg, or placebo once daily. Investigators were encouraged to follow local guidelines for achieving glycemic control by adjusting background glucose-lowering therapy as needed (after the initial 12 weeks of treatment where glucose-lowering treatment was to be kept unchanged), and for treating other CV risk factors. The primary endpoint was 3P-MACE, and the trial was designed to continue until at least 691 patients had experienced such an event.

This post hoc analysis included all trial participants treated with at least 1 dose of the study drug. We assigned participants to 3 categories according to their achievement of 0–3, 4–5, or 6–7 of the following 7 goals for CV risk factor control at baseline: (1) HbA1c <7.5%; (2) LDL cholesterol <100 mg/dL or statin use; (3) systolic blood pressure (SBP) <140 mmHg and diastolic blood pressure (DBP) <90 mmHg; (4) use of angiotensin-converting enzyme (ACE) inhibitor or angiotensin II receptor blocker; (5) normoalbuminuria; (6) aspirin use; and (7) nonsmoking status. Cox regression models were used to analyze the risk of CV death, hospitalization for heart failure, the composite of CV death (excluding fatal stroke) or hospitalization for heart failure, and 3P-MACE. We assessed the risk between the categories of risk factor goal achievement within the placebo group using a model with terms for age, sex, baseline BMI, baseline HbA1c, baseline eGFR, geographical region, and CV risk factor goal attainment at baseline. Using a second model, we assessed the risk with empagliflozin (both doses pooled) versus placebo across all CV risk factor goal-attainment categories and included the same terms as above plus additional terms for treatment and interaction of CV risk factor goal attainment at baseline and treatment. We performed sensitivity analyses using stricter goals for LDL cholesterol (<70 mg/dL or statin use) and SBP/DPB (<130/<80 mmHg). Another sensitivity analysis was undertaken excluding baseline HbA1c from the model, since this was 1 of the 7 risk factors investigated. Cardiovascular outcomes were also analyzed across CV risk factor goal attainment among men and women by using a model with terms for age, baseline BMI, baseline HbA1c, baseline eGFR, geographical region, treatment, sex/CV risk factor goal attainment at baseline, and interaction of treatment by sex/CV risk factor goal attainment at baseline.

Furthermore, we assessed the impact of the control of HbA1c (<7.5%), SBP (<140 mmHg), DBP (<90 mmHg), and LDL cholesterol (<100 mg/dL) during the trial, as time-dependent covariates on the treatment effect for outcomes using Cox regression models, with additional terms for age, sex, baseline BMI, baseline eGFR, treatment, geographic region, and baseline BP + LDL + HbA1c control.

Each patient who did not have an event was censored on the last day they were known to be free of the outcome. All analyses were performed on a nominal 2-sided α = 0.05, without adjustment for multiplicity.

## Results

### Baseline characteristics

**Figure 1.**
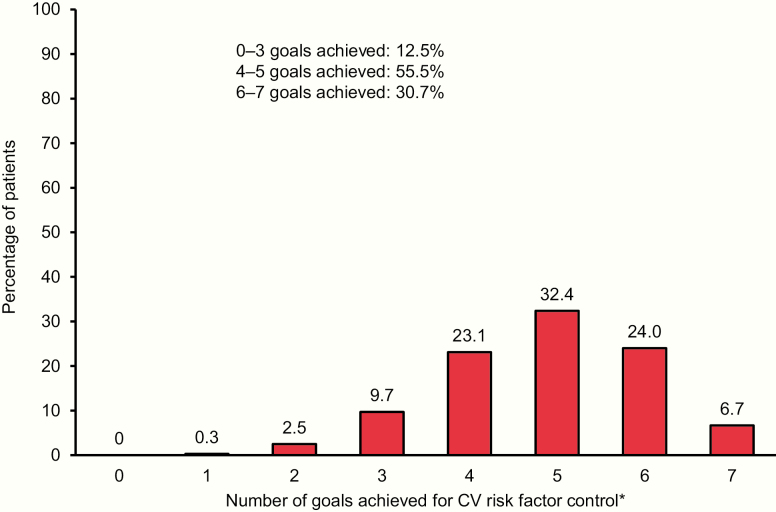
CV risk factor control at baseline in EMPA-REG OUTCOME trial. Data on attainment of CV risk factor goals at baseline were unavailable for 85 patients (66 empagliflozin and 19 placebo) (1.2%). *glycated hemoglobin <7.5%, low-density lipoprotein cholesterol <100 mg/dL or statin use, systolic blood pressure <140 and diastolic blood pressure <90 mmHg, use of angiotensin-converting enzyme inhibitor or angiotensin II receptor blocker, normoalbuminuria, nonsmoking, aspirin use.

The percentage of patients achieving the 7 goals for CV risk factor control is shown in Fig. 1. Only 1 patient did not achieve any of the 7 goals, while only 468 patients (6.7%) achieved all 7. The most common number of goals achieved was 5 (32.4% of patients). Baseline age, BMI, eGFR, and diabetes duration were similar in each category of CV risk factor goal achievement (Table 1). However, compared with the category achieving 6–7 risk factor goals at baseline, the category achieving only 0–3 goals had a higher prevalence of a history of stroke (30.7% vs 19.6%) and a lower prevalence of coronary artery disease (60.3% vs 82.7%) and history of MI (37.8% vs 50.8%). By definition, HbA1c, SBP, DBP, LDL cholesterol, use of statins, albuminuria, smoking status, and use of ACE inhibitors or angiotensin II receptor blockers varied between categories.

**Table 1.** Baseline characteristics of patients by achievement of CV risk factor goals at baseline: HbA1c <7.5%; LDL cholesterol <100 mg/dL or statin use; SBP <140 mmHg and DBP <90 mmHg; use of ACE inhibitor or ARB; normoalbuminuria; aspirin use; and/or nonsmoking.

	CV Risk Factor Goals Attained at Baseline		
	0−3 (n = 884)	4−5 (n = 3895)	6−7 (n = 2156)
Male	611 (69.1)	2773 (71.2)	1567 (72.7)
Age, years	62.5 ± 9.3	63.4 ± 8.6	63.1 ± 8.4
Race			
White	620 (70.1)	2814 (72.2)	1574 (73.0)
Asian	211 (23.9)	854 (21.9)	442 (20.5)
Black/African-American	46 (5.2)	194 (5.0)	115 (5.3)
American Indian/Alaska Native	7 (0.8)	27 (0.7)	20 (0.9)
Native Hawaiian/Other Pacific Islander	0	5 (0.1)	5 (0.2)
Region			
Europe	405 (45.8)	1647 (42.3)	786 (36.5)
Asia	191 (21.6)	773 (19.8)	381 (17.7)
North America	133 (15.0)	707 (18.2)	536 (24.9)
Latin America	124 (14.0)	603 (15.5)	342 (15.9)
Africa	31 (3.5)	165 (4.2)	111 (5.1)
BMI, kg/m^2^	30.24 ± 5.38	30.58 ± 5.21	30.81 ± 5.30
HbA1c, %	8.38 ± 0.79	8.19 ± 0.82	7.72 ± 0.80
Time since T2DM diagnosis, years			
>10	499 (56.4)	2323 (59.6)	1147 (53.2)
>5 to 10	217 (24.5)	937 (24.1)	564 (26.2)
>1 to 5	153 (17.3)	548 (14.1)	368 (17.1)
≤1 year	15 (1.7)	87 (2.2)	77 (3.6)
Glucose-lowering therapy			
Metformin	620 (70.1)	2873 (73.8)	1640 (76.1)
Sulfonylurea	415 (46.9)	1649 (42.3)	906 (42.0)
Insulin	420 (47.5)	1974 (50.7)	952 (44.2)
eGFR (MDRD), mL/min/1.73 m^2^	75.39 ± 24.06	73.63 ± 21.15	74.16 ± 20.67
≥90	226 (25.6)	846 (21.7)	445 (20.6)
60 to <90	418 (47.3)	2019 (51.8)	1181 (54.8)
<60	240 (27.1)	1029 (26.4)	530 (24.6)
UACR, mg/g			
<30	198 (22.4)	1954 (50.2)	2006 (93.0)
30−300	467 (52.8)	1426 (36.6)	116 (5.4)
>300	219 (24.8)	515 (13.2)	34 (1.6)
Current smoker	300 (33.9)	543 (13.9)	71 (3.3)
Any CV disease			
Coronary artery disease	533 (60.3)	2929 (75.2)	1784 (82.7)
History of myocardial infarction	334 (37.8)	1811 (46.5)	1095 (50.8)
History of stroke	271 (30.7)	922 (23.7)	422 (19.6)
Heart failure	83 (9.4)	397 (10.2)	222 (10.3)
Systolic blood pressure, mmHg	146.4 ± 17.6	137.6 ± 16.9	126.9 ± 12.7
Diastolic blood pressure, mmHg	81.2 ± 10.4	77.2 ± 9.7	73.8 ± 8.9
LDL cholesterol, mg/dL	108.2 ± 40.9	85.2 ± 35.0	76.7 ± 30.4
Antithrombotics			
Aspirin	450 (50.9)	3191 (81.9)	2091 (97.0)
Clopidogrel	85 (9.6)	427 (11.0)	224 (10.4)
Vitamin K antagonists	70 (7.9)	261 (6.7)	86 (4.0)
Statins	410 (46.4)	3056 (78.5)	1889 (87.6)
Antihypertensives			
ACE inhibitors/ARBs	436 (49.3)	3102 (79.6)	2060 (95.5)
β-blockers	456 (51.6)	2527 (64.9)	1515 (70.3)
Diuretics	310 (35.1)	1668 (42.8)	1019 (47.3)
Calcium channel blockers	297 (33.6)	1344 (34.5)	654 (30.3)

Data are n (%) or mean ± SD in patients treated with ≥1 dose of the study drug in the pooled empagliflozin and placebo treatment groups. Data on attainment of CV risk factor goals at baseline were unavailable for 85 patients (66 empagliflozin and 19 placebo).

Abbreviations: ACE, angiotensin-converting enzyme; ARB, angiotensin II receptor blocker; BMI, body mass index; CV, cardiovascular; eGFR, estimated glomerular filtration rate; HbA1c, glycated hemoglobin; LDL, low-density lipoprotein; MDRD, modification of diet in renal disease; T2DM, type 2 diabetes mellitus; UACR, urine albumin-to-creatinine ratio.

### CV outcomes

**Figure 2.**
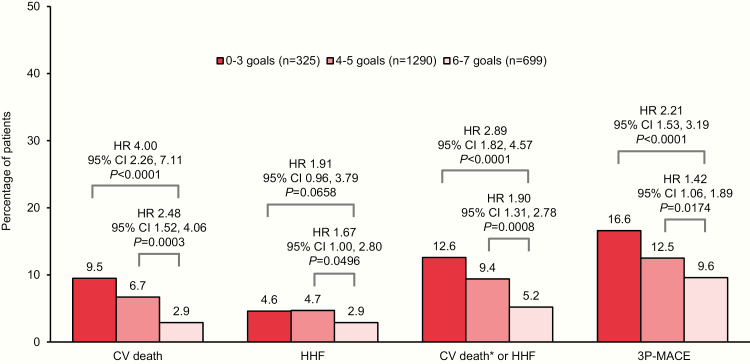
Risk of CV outcomes in the placebo group by achievement of CV risk factor goals at baseline: HbA1c <7.5%; LDL cholesterol <100 mg/dL or statin use; SBP <140 mmHg and DBP <90 mmHg; use of ACE inhibitor or ARB; normoalbuminuria; aspirin use; and/or nonsmoking. Cox regression analysis in patients treated with ≥1 dose of the study drug. Data on attainment of CV risk factor goals at baseline were unavailable for 85 patients (66 empagliflozin and 19 placebo). 3P-MACE indicates 3-point major adverse CV events (CV death, nonfatal myocardial infarction, or nonfatal stroke). Abbreviations: ACE, angiotensin-converting enzyme; ARB, angiotensin II receptor blocker; CV, cardiovascular; DBP, diastolic blood pressure; HbA1c, glycated hemoglobin; HHF, hospitalization for heart failure; HR, hazard ratio; LDL, low-density lipoprotein; SBP, systolic blood pressure. *Excludes fatal stroke.

The median observation time in the EMPA-REG OUTCOME trial was 3.1 years (28). In the placebo group, participants achieving only 0–3 or 4–5 risk factor goals at baseline had a significantly increased risk for subsequent CV death, the composite of CV death (excluding fatal stroke) or hospitalization for heart failure, and 3P-MACE, compared with those achieving 6–7 goals (Fig. 2). For hospitalization for heart failure, although pointing in the same direction, there was only a small increase in the point estimate (hazard ratio) with 0–3 and 4–5 versus 6–7 goals, and only the latter was significant (Fig. 2). The sensitivity analysis with stricter goals for LDL cholesterol, SBP, and DBP showed similar results with significant risk differences between those achieving only 0–3 compared to 6–7 goals for CV death, CV death or hospitalization for heart failure, and 3P-MACE, but not for hospitalization for heart failure alone (data not shown). The sensitivity analysis without adjusting for baseline HbA1c showed very similar results to the main model (data not shown).

**Figure 3.**
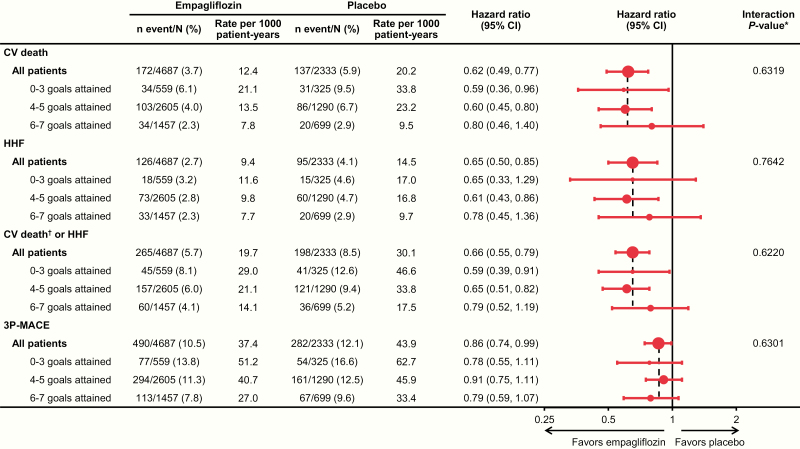
Effect of empagliflozin versus placebo on CV outcomes across subgroups of achievement of CV risk factor goals at baseline: HbA1c <7.5%; LDL cholesterol <100 mg/dL or statin use; SBP <140 mmHg and DBP <90 mmHg; use of ACE inhibitor or ARB; normoalbuminuria; aspirin use; and/or nonsmoking. Cox regression analysis in patients treated with ≥1 dose of the study drug. Data on attainment of CV risk factor goals at baseline were unavailable for 85 patients (66 empagliflozin and 19 placebo). 3P-MACE indicates 3-point major adverse CV events (CV death, nonfatal myocardial infarction, or nonfatal stroke). Abbreviations: ACE, angiotensin-converting enzyme; ARB, angiotensin II receptor blocker; CI, confidence interval; CV, cardiovascular; DBP, diastolic blood pressure; HbA1c, glycated hemoglobin; HHF, hospitalization for heart failure; LDL, low-density lipoprotein; SBP, systolic blood pressure. *P-value relates to test of homogeneity of treatment group differences among subgroups (test for treatment by subgroup interaction) without adjustment for multiple testing. ^†^Excludes fatal stroke.

The treatment effect of empagliflozin in reducing risk for CV death, hospitalization for heart failure, the composite of CV death (excluding fatal stroke) or hospitalization for heart failure, and 3P-MACE was consistent across all categories of risk factor control at baseline (*P* > 0.05 for all tests for interaction between treatment and CV risk factor goal attainment at baseline) (Fig. 3).

Similar findings were seen in sensitivity analysis that included stricter treatment goals for LDL cholesterol (<70 mg/dL or statin use), SBP (<130 mmHg), and DBP (<80 mmHg) (data not shown). Again, empagliflozin consistently lowered the risk of CV events versus placebo across the 3 categories of goal achievement.

The additional sensitivity analysis used the less strict goals for LDL cholesterol (<100 mg/dL) and blood pressure (SBP/DBP <140/90) but did not include a term for baseline HbA1c in the regression model. Here, again, the findings were very similar, showing a consistent treatment effect of empagliflozin across all categories of goal achievement at baseline (data not shown).

Empagliflozin consistently reduced the risk for CV events across the different categories of risk factor control at baseline by sex (all interactions, *P* > 0.05 [data not shown]). The sensitivity analysis with stricter goals for LDL cholesterol and blood pressure showed similar results (data not shown).

**Figure 4.**
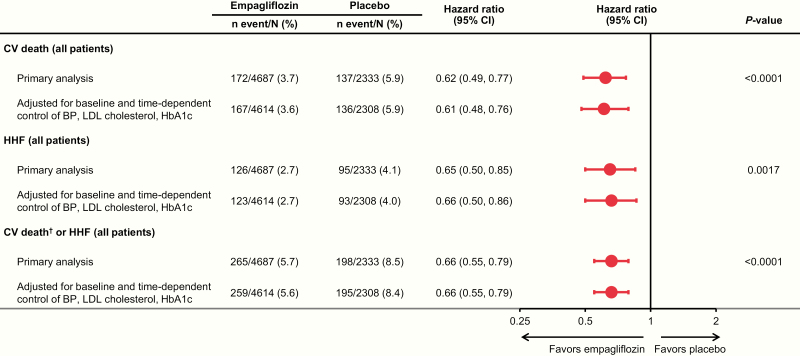
Effect of empagliflozin versus placebo on CV outcomes overall and adjusted for control of HbA1c (<7.5%), SBP (<140 mmHg), DBP (<90 mmHg), and LDL cholesterol (<100 mg/dL) during the trial. Cox regression analysis in patients treated with ≥1 dose of the study drug. Abbreviations: BP, blood pressure; CI, confidence interval; CV, cardiovascular; DBP, diastolic blood pressure; HbA1c, glycated hemoglobin; HHF, hospitalization for heart failure; LDL, low-density lipoprotein; SBP, systolic blood pressure. ^†^Excludes fatal stroke.

The analyses adjusting for control of HbA1c, SBP, DBP, and LDL cholesterol during the trial showed consistent reductions in outcomes with empagliflozin versus placebo (Fig. 4).

## Discussion

Patients with T2D and established ASCVD are at very high risk for CV events (1, 2). This post hoc analysis of the landmark EMPA-REG OUTCOME trial found that control of more versus fewer CV risk factors in such individuals was associated with a lower risk for subsequent CV events and mortality, although not significantly for all comparisons. Furthermore, treatment with the SGLT2 inhibitor empagliflozin was associated with reduced risk of CV death, hospitalization for heart failure, CV death or hospitalization for heart failure, and 3P-MACE versus placebo regardless of the level of CV risk factor control at baseline.

These findings add to the body of evidence that simultaneous control of multiple CV risk factors reduces the risk of ASCVD in T2D. Although seemingly self-evident, data to support this hypothesis are surprisingly sparse. One of the most compelling studies was the Steno-2 randomized trial, which commenced in 1993 (7). Multifactorial intensive treatment of hyperglycemia, hypertension, dyslipidemia, and microalbuminuria in patients with type 2 diabetes and microalbuminuria halved their risk of atherosclerotic CV events at a mean follow-up of 7.8 years, compared with conventional treatment (7). Furthermore, although randomized treatment ended after 8 years, with all surviving participants subsequently receiving intensive therapy, there was a sustained reduction in CV risk and mortality as well as for hospitalizations for heart failure for 21 years in the group originally randomized to intensive treatment, with a median 7.9 years of life gained (31–34). Although these findings were notable (35), the impact of Steno-2 was limited by its single-center design, small and ethnically homogenous cohort (160 Northern European white patients), and nonblinded treatment allocation in the prestatin era. More recently, the J-DOIT3 multicenter randomized trial in Japan evaluated intensive multifactorial treatment targeting hyperglycemia, hypertension, and dyslipidemia. This strategy did not significantly reduce the risk of overall atherosclerotic CV events after a median 8.5 years of follow-up as compared with standard care, although the incidence of stroke was halved (9). Both Steno-2 and J-DOIT3 were conducted in patients with longstanding T2D. In contrast, the ADDITION-Europe randomized trial enrolled patients with T2D immediately following their diagnosis by screening. The intensive multifactorial regimen employed in this study targeted glycemic control, hypertension, dyslipidemia and aspirin prophylaxis, and was associated with a non-significant 17% reduction in risk of ASCVD events after a mean follow-up of 5.3 years (8). Furthermore, unlike Steno-2, there was no legacy effect of treatment in terms of CV risk reduction after a further 5 years postintervention (mean duration of follow-up of 9.6 years) (36).

Other studies supporting multifactorial intervention for CV risk reduction in T2D are largely observational in nature (10–19). A post hoc analysis of the multinational BARI-2D trial (2001–2008) in T2D patients with stable coronary artery disease found that the more risk factors controlled during the trial (HbA1c, blood pressure, non-HDL cholesterol, triglycerides, nonsmoking), the lower the risk of all-cause mortality and major atherosclerotic CV events over the following 5 years (15). Interestingly, unlike the current analysis of the EMPA-REG OUTCOME trial, there was no relationship between the number of risk factors controlled at baseline and the risk for subsequent CV events or death (15). In a cohort study of over 200 000 T2D patients in the Swedish National Diabetes Register from 1998 to 2012, there was a step-wise decrease in excess risk for each risk factor that was controlled, and those with control of 5 risk factors (HbA1c, LDL cholesterol, blood pressure, albuminuria, smoking) had little to no increased risk for mortality, MI, or stroke over the subsequent 6 years compared with the general population (17). Cohort studies of the Kaiser Permanente Northwest diabetes registry (14) and T2D patients with chronic kidney disease in the United Kingdom (16) also found reduced risk of ASCVD events with simultaneous control of CV risk factors. A recent post hoc analysis of the TECOS CV outcomes trial of the dipeptidyl peptidase-4 inhibitor sitagliptin found that attainment of 5 risk factor measures at baseline (aspirin use, LDL cholesterol <70 mg/dL or statin use, SBP/DBP <140/<90 mmHg, use of ACE inhibitor or angiotensin II receptor blocker, nonsmoking) was associated with reduced risk for CV death, MI, and stroke in T2D patients with established CV disease (18). However, this study did not explore the effects of sitagliptin versus placebo by baseline risk factor control. Finally, the MEMO study in the United Kingdom found that multifactorial intervention with structured diabetes self‐management education benefited cardiometabolic risk factor profiles compared with usual diabetes care. Furthermore, despite intensive glycemic control, there was no increase in severe hypoglycemia or CV death, albeit these 2 outcomes were not powered (37).

The current analysis also suggests that simultaneous control of multiple CV risk factors may reduce the risk for heart failure hospitalizations as well as ASCVD in patients with T2D. Although T2D was long thought to be a coronary heart disease risk equivalent (4), more recent studies suggest a lower magnitude of risk in the modern era of CVD prevention (38, 39). In contrast, the magnitude of risk for heart failure in T2D was underappreciated until recently and may be equal to or even greater than the risk for atherosclerotic events (20, 22). For example, an observational study of a large US claims database found that insulin-treated T2D patients had hospitalization rates per 10 000 patient years of 97 and 151 for MI and stroke, respectively, compared with 243 for heart failure (21). Furthermore, heart failure is the 1 CV outcome for which glucose-lowering drugs, depending on type, have been shown to both increase risk (thiazolidinediones (40, 41), the dipeptidyl peptidase-4 inhibitor saxagliptin (42)) and decrease risk (SGLT2 inhibitors (43)). Interestingly, in the study of T2D patients in the Swedish National Register, control of all 5 risk factors reduced but did not eliminate the excess risk of hospitalization for heart failure compared with controls without diabetes (17). Our results are in line with this, showing that the risk of heart failure hospitalization was not as closely associated with the number of risk factors controlled. This is supported by a mechanistic study showing no impact on cardiac function of a 2-year multifactorial intervention targeting lifestyle intervention, hyperglycemia, hypertension, and dyslipidemia in T2D patients (44). These findings can be explained by potential nontraditional, unmeasured risk factors that play a bigger role in heart failure events than atherosclerotic outcomes (44). As neither glucose-lowering (45) nor lipid-lowering (46) per se have shown treatment benefit for heart failure, therapeutic options are limited.

The other main finding of the current analysis is that the cardioprotective effect of empagliflozin was evident regardless of the number of risk factors controlled at baseline in both women and men. Thus, even in patients with well-controlled traditional CV risk factors, the risk for CV death, hospitalization for heart failure, CV death or hospitalization for heart failure, and 3P-MACE associated with T2D is reduced with empagliflozin. This is, moreover, consistent with previous subgroup analyses of the EMPA-REG OUTCOME trial, which found CV risk reduction regardless of prior coronary artery bypass graft (47, 48), peripheral artery disease (48, 49), heart failure (48, 50), atrial fibrillation (48), chronic kidney disease (51), microvascular disease (52), the risk of CV disease (53) or heart failure (54), age (55), sex (29), glycemic control (56), or incident hypoglycemia (57). Thus, CV risk reduction with empagliflozin does not seem to be affected by the presence of CV comorbidities or classic risk factors. Moreover, the CV benefit with empagliflozin was also evident irrespective of control of HbA1c, SBP, DBP, and LDL cholesterol during the trial, emphasizing that empagliflozin offers CV protection, which is additive to the benefits of controlling conventional CV risk factors.

The mechanism for this reduction in CV risk has not been clearly defined. The pharmacodynamic effect of empagliflozin is to increase glucosuria and natriuresis (58, 59), achieved via inhibition of the SGLT2 transporter in the proximal tubule of the kidney. This leads to reductions in plasma volume (60), increases in hematocrit (61), and decreases in arterial stiffness and vascular resistance (62). An exploratory mediation analysis of the EMPA-REG OUTCOME trial suggested that changes in markers of plasma volume were the most important mediators of the reduction in CV deaths (61). Other hypotheses proposed to explain the cardioprotective effects of SGLT2 inhibitors include improved myocardial bioenergetics via a shift in fuel source from glucose to ketone bodies, inhibition of the cardiomyocyte sodium-hydrogen exchanger, and antifibrotic effects in the heart (63, 64). The fact that these proposed mechanisms do not involve conventional CV risk factors might explain the consistent treatment effect of empagliflozin in our analysis. Incidentally, a recent analysis from the EMPA-REG OUTCOME trial showed that changes in classic risk factors appeared to explain only a small proportion of the CV effects observed (65). The recent DAPA-HF trial suggests that glucose-lowering plays little part in the amelioration of heart failure, at least, by SGLT2 inhibitors, as almost identical risk reductions for heart failure were seen in individuals with and without T2D (66).

Finally, our analysis reinforces recent studies showing that a substantial number of people with T2D globally do not meet treatment goals for controlling CV risk factors (14, 17, 18, 67–69). The majority of patients in the EMPA-REG OUTCOME trial (56%) had achieved only 4 or 5 goals at study entry, less than a third (31%) had met 6 or 7, and only 6.7% had achieved all 7 goals—and there were no substantial differences between women and men. Interestingly, we observed that among those with fewer CV risk factors controlled in the EMPA-REG OUTCOME trial, there was a higher proportion of patients with a prior stroke, whereas among those with more risk factors controlled, there were more with coronary artery disease. This may suggest differential risk factor goal attainment depending on the vascular bed in which ASCVD is manifest, consistent with a previous report in an ambulatory population with coronary artery disease, cerebrovascular disease, or both, 37% of whom had diabetes (70).

Our analysis has some limitations, including omission of other, nontraditional, risk factors for ASCVD such as inflammation, genetic factors, and socioeconomic status that were not captured during the trial. Furthermore, CV risk factors were analyzed categorically rather than continuously; however, this has the advantage of being pragmatic for use in routine clinical practice. Also, the data are derived from a population with established ASCVD and so may not extend to all T2D patients. These limitations must be balanced against the strengths of the analysis, notably that data are derived from a high-quality, event-driven, randomized clinical trial in which CV and heart failure outcomes were adjudicated by independent committees.

In conclusion, the risk for CV events increased with the decreasing number of CV risk factors controlled at baseline in the EMPA-REG OUTCOME trial, but the cardioprotective effect of empagliflozin was consistent regardless of the level of risk factor control.

## Additional Information

***Disclosure Summary:*** S.E.I. has consulted and/or served on clinical trial steering/executive/publications committees for Boehringer Ingelheim (BI), AstraZeneca, Novo Nordisk, Sanofi/Lexicon Pharmaceuticals, Merck, vTv Therapeutics, and Abbott/Alere. K.K. has received research grants and acted as a consultant, advisory board member, and speaker for AstraZeneca, BI, Bristol-Myers Squibb, Berlin-Chemie AG/Menarini Group, Eli Lilly and Company, Merck Sharp & Dohme, Novo Nordisk, Roche, and Sanofi. D.H.F. has received honoraria from Amgen, AstraZeneca, BI, Eli Lilly and Company, Merck & Co., and Sanofi. C.W. has received honoraria for consultancy and lecturing from Abbvie, Actelion, Amgen, Bayer, BI, GlaxoSmithKline, Janssen, Eli Lilly and Company, Protalix, Sanofi Genzyme, and Shire. M.M., J.T.G., and A.P.O. are employees of BI. B.Z. has received research grants awarded to his institution from BI, AstraZeneca, and Novo Nordisk, and honoraria from Janssen, Sanofi, Eli Lilly and Company, BI, Novo Nordisk, and Merck Sharp & Dohme.

***Data Availability:*** The sponsor of the EMPA-REG OUTCOME trial (Boehringer Ingelheim) is committed to responsible sharing of clinical study reports, related clinical documents, and patient-level clinical study data. Researchers are invited to submit inquiries via the Vivli website (https://www.vivli.org).
